# Immobilization of Electroporated Cells for Fabrication of Cellular Biosensors: Physiological Effects of the Shape of Calcium Alginate Matrices and Foetal Calf Serum

**DOI:** 10.3390/s90100378

**Published:** 2009-01-09

**Authors:** Nikos Katsanakis, Andreas Katsivelis, Spiridon Kintzios

**Affiliations:** 1 Laboratory of Plant Physiology, Faculty of Agricultural Biotechnology, Agricultural University of Athens, Greece; 2 EMBIO Diagnostics Project, Nicosia, Cyprus

**Keywords:** Calcium alginate, Cell immobilization, Electroporation, Foetal calf serum (FCS), Monkey African green kidney (Vero) cells

## Abstract

In order to investigate the physiological effect of transfected cell immobilization in calcium alginate gels, we immobilized electroporated Vero cells in gels shaped either as spherical beads or as thin membrane layers. In addition, we investigated whether serum addition had a positive effect on cell proliferation and viability in either gel configuration. The gels were stored for four weeks in a medium supplemented or not with 20% (v/v) foetal calf serum. Throughout a culture period of four weeks, cell proliferation and cell viability were assayed by optical microscopy after provision of Trypan Blue. Non-elaborate culture conditions (room temperature, non-CO_2_ enriched culture atmosphere) were applied throughout the experimental period in order to evaluate cell viability under less than optimal storage conditions. Immobilization of electroporated cells was associated with an initially reduced cell viability, which was gradually increased. Immobilization was associated with maintenance of cell growth for the duration of the experimental period, whereas electroporated cells essentially died after a week in suspension culture. Considerable proliferation of immobilized cells was observed in spherical alginate beads. In both gel configurations, addition of serum was associated with increased cell proliferation. The results of the present study could contribute to an improvement of the storability of biosensors based on electroporated, genetically or membrane-engineered cells.

## 1. Introduction

Electroporation is a standard method for the production of specifically responding cells, either through genetic engineering [1] or through the electroinsertion of receptor-like molecules on the cell membrane (membrane-engineering) [2, 3]. The utility of electroporation for incorporation of exogenous molecules into living cells depends upon the formation of a permeable state in the cell membrane, which in turn depends upon the field strength and duration of the applied electric pulse. Frequently, high field strengths (required by various experimental protocols) are associated with increased cell death. Therefore it is highly desired to apply any conditions contributing to increasing the viability of electroporated cells. Bahnson and Boggs [4] reported that serum contains components which rapidly reseal the membranes of electroporated murine myelomonocytic leukaemia cells, therefore improving survival of highly permeated cells and increasing the DNA electrotransfection efficiency. Delteil *et al.* [5] demonstrated a concentration-dependent increase in electroporated cell viability: the number of surviving Chinese hamster ovary (CHO) cells after administration of 20% (v/v) FCS was almost three times higher than control.

The successful application of an immobilized cell system as a biocatalyst relies on the proper choice of the main components of the system, including the matrix material, the configuration of the immobilization system, the cell type and the formulation of the nutrient medium. In the case of cellular biosensors based on immobilized cells, immobilization not only helps in forming the required close proximity between the biomaterial and the transducer, but also helps in stabilizing it for reuse [6, 7]. Among the desirable characteristics for immobilized cell systems are a high surface area-to-volume ratio, chemical and mechanical stability and optimum diffusion of oxygen and nutrients [8].

Various studies have demonstrated that the physiology of immobilized cells is generally comparable to cells in suspension [7]. The majority of these experiments have been carried out using bacterial cells, while only a few reports are focused on the properties of immobilized mammalian cells [9, 10]. In these few cases, immobilization was found to considerably affect cell metabolism and cell apoptosis, possibly due to changes in local nutrient concentration as a result of mass transfer limitations [11].

Calcium alginate is a very favorable immobilization matrix for living cells, due to its low cost, wide availability and very mild gelling conditions [12]. In a recent report [13], we investigated the effect of the shape of the immobilization matrix on the viability and physiology of Vero cells entrapped in calcium alginate gels shaped either as spherical beads or as thin membrane layers. Vero cell proliferation was observed only in spherical beads and was associated with increased [Ca^2+^]_cyt_., as well as increased mass and oxygen transfer from the surrounding nutrient medium to the immobilized cells.

Therefore, in the present study we investigated the effect of both the shape of the calcium alginate matrix and foetal calf serum addition on the viability and proliferation of electroporated Vero cells. The results of the study are discussed in view of their practical implementation in the construction of cell biosensors.

## 2. Experimental Section

### 2.1. Cell culture

Monkey African green kidney (Vero) cells were routinely subcultured as described previously [8, 9]. Prior to immobilization, cells were clonally expanded by subculture in Dulbecco's medium with 10% heat-inactivated bovine serum, 10% antibiotics (streptomycin) and 10% l-glutamine. Each subculture lasted 3 days and cells were trypsinized at a 1:3 ratio. After cell detachment from the culture vessel by adding trypsin/EDTA for 10 min at 37 °C and cell concentration by centrifugation (6 min, 1,200 rpm, 25 °C), cells were ready for electropulsation and immobilization according to the following protocols.

#### 2.1.1. Electroporation

The electroporation protocol has been described elsewhere [2, 14]. Briefly, cells were centrifuged for 6 min at 1,000 rpm and resuspended in the ‘pulsing buffer’ at a concentration of 3×10^6^ cells per mL. The volume of the pulsing buffer was 200 μL textural of 0.14 M NaCl, 5 mM NaH_2_PO_4_H_2_O, 5 mM Na_2_HPO_4_12H_2_O, pH 7.4. The low ionic content saline buffer allowed for the delivery of pulses of long duration (ms) at high voltages (1,800 V). The solution was transferred to the specific electroporation tube of the voltage generator (EC100, Thermo) which gave square-wave electric pulses. The experiments were performed under a laminar flow hood. Two pulses lasting 5 ms were applied. Control electroporated cells were not immobilized and cultured in suspension.

#### 2.1.2. Cell immobilization

- Ca–Alginate Thin Layer (TL). Following a modified procedure of the method described by De Backer *et al.* [15] and Kintzios *et al.* [10], one milliliter of cell suspension (electroporated or control) was mixed with 4% (w/v) sodium alginate solution (3 mL), then transferred on a filter paper (with a 5 cm diameter) soaked in a Petri dish in 0.8 M CaCl_2_ (1.5 mL). The resulting calcium alginate gel membrane, with dimensions 4 mm × 6 mm × 0.5 mm and containing cells, was solidified within 2–3 min and was 1 mm thick.
- Ca–Alginate Spherical Beads (SB). cell suspension (electroporated or control, 1 mL) was mixed with 4% (w/v) sodium alginate solution (3 mL) and then the mixture was added dropwise, by means of a 22G syringe, to 0.8 M CaCl_2_. Each of the resulting calcium alginate beads had an approximate diameter of 2.5–3 mm.

#### 2.1.3. Cell storage in alginate gels

Approximately 50,000 cells were immobilized per gel bead or thin layer. Gels with immobilized cells were maintained in Dulbecco's medium with 20% (v/v) foetal calf serum (FCS) or without FCS, at room temperature (24±0.3°C), under normal atmospheric (i.e. non-CO_2_-enriched) conditions.

### 2.2. Optical microscopy assays

Thin cross-sections (approximately 10 μm thick) of gels with immobilized cells were placed on a haemocytometer, mounted on an optical microscope with a digital camera (SONY S75 digital still camera) attached to it and trypan blue (10 μL) was added for 5 minutes at room temperature to each configuration. The number of viable (unstained) and dead (stained) cells was counted. The average number of unstained cells in each quadrant was calculated to find the density of cells per optical field and the percentage of cell death was extracted. Five cross-sections were made from each gel bead or a gel thin layer. The orientation of each cross-section was random, so that the totality of all cross-sections would equally represent cell populations in different locations within the gel. Cell proliferation was assayed by means of simple cell counting, without the addition of any dye. The number of cells (stained or not) was counted both manually and by using Doc-It^®^LS Image Acquisition and Analysis Software.

### 2.3. Chemicals

Unless otherwise stated, all solvents and chemicals used were of analytical quality and provided from Fluka. Water was double distilled. Cell cultures were originally provided from the American Type Culture Collection (ATCC).

## 3. Results and Discussion

**Figure 1.**
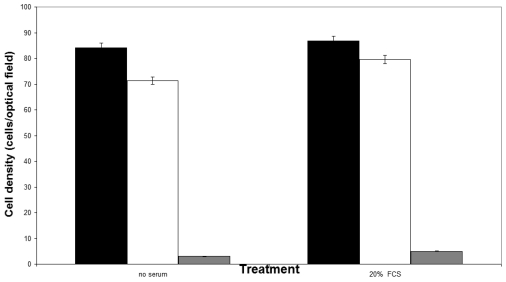
Effect of supplementation of 20% (v/v) FCS to the nutrient medium on the proliferation of electroporated Vero cells *in suspension* (black columns: day 1, white columns: day 2, gray columns: day 7) (*n* = 10 replications and error bars represent standard errors of the average value of all replications).

**Figure 2.**
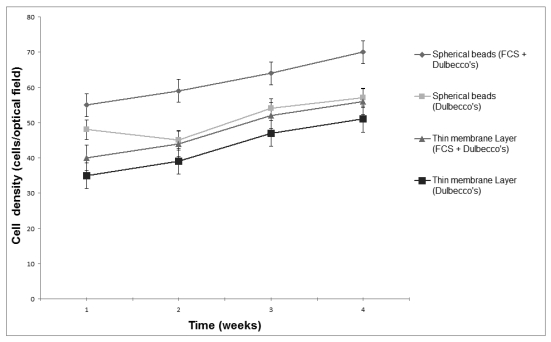
Effect of the shape of the immobilization matrix and the addition of 20% (v/v) FCS on the proliferation of electroporated Vero cells (electroinsertion of SOD, *n* = 10 replications and error bars represent standard errors of the weekly average value of all replications).

**Figure 3.**
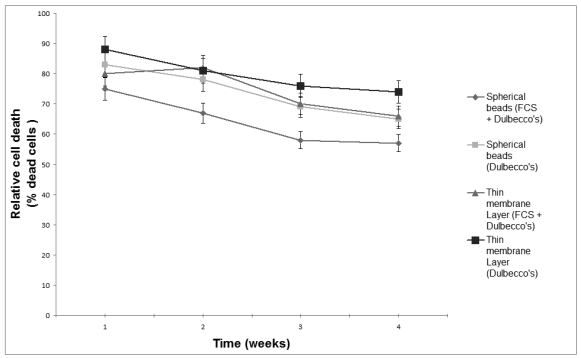
Effect of the shape of the immobilization matrix and the addition of 20% (v/v) FCS on the death of electroporated Vero cells, as indicated by trypan blue staining (electroinsertion of SOD. Percentage of dead cells which absorb the stain/cell density. *n*=10 replications and error bars represent standard errors of the weekly average value of all replications).

Although more than 80% of the electroporated Vero cells were viable one day after electroporation, less than 3% survived in suspension after a week (Figure 1). Supplementation of the culture medium with 20% (w/v) FCS was associated with a slight increase in the number of viable cells. On the contrary, immobilization of electroporated cells was associated with an initially decreased cell viability, which was gradually increased over a four-week culture period. Both the addition of FCS and the shape of the immobilization matrix had a considerable effect on cell proliferation. During the four-week culture period, considerable proliferation of immobilized cells was observed in spherical alginate beads, especially when the nutrient medium was supplemented with FCS, while the observed cell proliferation in thin layer membrane was lower (Figure 2). In both gel configurations, addition of FCS was associated with increased cell proliferation. Immobilization effects on cell proliferation were inversely associated with the observed patterns of cell death: in spite of the high occurrence of dead cells at the beginning of the culture (75–88%, depending on treatment), the percentage of dead (Trypan Blue-stained) cells in all configurations declined during the culture period (Figure 3).

Increased cell proliferation in spherical beads, compared to thin gel layers, was previously reported for non-electroporated Vero cells [16]. This “gel-shape” effect was attributed to an increased efficiency of mass transfer from the nutrient medium to the immobilization matrix, which was estimated by calculating the diffrerent mass transfer Biot number (Bi_m_) of spherical beads and thin layers. Immobilization effects on cell proliferation were invertedly associated with cell death and [Ca^2+^]_cyt_ accummulation. Other reports demonstrated the beneficial effect of immobilization in 3-D matrices on cell growth and differentiation [13, 17, 18]. Nevertheless, the immobilization procedure itself can result in considerable cell stress, thus inhibiting cell division [19], frequently accompanied by increased oxidative stress [20] or even leading to cell death [16]. This could provide an explanation for the observed initially lower viability of immobilized cells compared to cells cultured in suspension.

There are only a few reports on the beneficial effect of serum on the survival of electroporated cells, whereas minimal side effects (concerning cell permeabilization to electroloaded molecules) have been reported. Delteil *et al.* [5] showed that cell viability was increased if cells were treated with serum before electroporation and the electroloaded molecules were added after serum in the electroporation medium. In the present study, cells were treated with serum after their electroporation and immobilization, therefore the possibility exists for increasing cell viability by adding serum to “pulsing electroporation buffer” (see 2.1.1. above). This approach is currently under investigation by our research group.

## 4. Conclusions

Cell immobilization in alginate gels is becoming quite popular in the fabrication of cell-based biosensors, while cell electroporation is a standard method for (especially mammalian) cell transfection in order to approach a desired level of recognition selectivity. From a practical point of view, the present study demonstrated that a combination of both techniques could provide an attractive solution for fabricating sensors with increased performance characteristics, including storability and operational stability. This strategy seems to have a broader applicability beyond Vero cells, as indicated by our yet unpublished results with a larger number of cell lines (lamp testis, neuroblastoma N2a and hamster adult kidney) immobilized in a variety of gelling agents (Ca-alginate, bactoagar, ι-carrageenan), whereas significant interactive (cell line X gelling agent) effects were observed. Further improvements in this direction could result from modifications of the physical or chemical sensor enviroment, such as the addition of serum or specific ions (K^+^, Mg^2+^) at high concentrations [21].
